# Macrophages in the Glioblastoma Tumor Microenvironment

**DOI:** 10.3390/ijms24108978

**Published:** 2023-05-19

**Authors:** Salvatore J. Coniglio

**Affiliations:** Department of Biological Sciences, School of Integrative Science and Technology, Kean University, Union, NJ 07083, USA; coniglsa@kean.edu

## Introduction

The prognosis of high-grade glioma remains dismal, with the median survival time being 15 months. The tumor microenvironment is the central driver of glioma malignancy. As is the case with most solid tumors, tumor-associated macrophages and other myeloid cells (TAMs) are the most abundant non-cancerous cell type within the glioma microenvironment. A substantial body of evidence has confirmed that TAMs contribute to glioma invasion, chemoresistance and immunosuppression. New therapies for high-grade glioma are urgently needed, and targeting TAMs within the glioma microenvironment could be a very fruitful approach.

The molecular ligands and receptors involved in mediating the paracrine interaction between glioma cells and TAMs have been investigated using in vitro and in vivo models. The expression of growth factors which act on macrophages such as Colony-Stimulating Factor-1 (CSF-1; also called M-CSF) and Granulocyte-Macrophage Colony-Stimulating Factor (GM-CSF) have been shown to be upregulated in high-grade gliomas and can mediate many of the pro-tumorigenic functions of TAMs. The inhibition of CSF-1 and its receptor, CSF-1R, in rodent models of glioma has been very effective. In some cases, the inhibition resulted in the complete reversal of tumor growth, likely due to the elicitation of an immunological response. However, the efficacy of anti-CSF-1 therapy in human clinical trials has shown very modest results. The search continues for other ligand/receptor pairs which mediate the malignant switch within the microenvironment. 

With the advent of immune checkpoint antibodies, chimeric antigen receptor (CAR)-T cell therapy, and other immunotherapies, the reversal of TAM-mediated immunosuppression could potentially yield great benefits. Interventions which promote an immunologically “hot tumor” could render glioma tumors much more sensitive to immunotherapy. Although it is promising, challenges lay ahead for this approach, as small-molecule inhibitors need to specifically target TAMs while sparing normal physiological myelopoiesis. 

This special edition of *International Journal of Molecular Sciences*, entitled “Macrophages in the Glioblastoma Tumor Microenvironment”, contains seven contributions including five review articles and two research articles. These papers cover some of the latest developments in glioma and TAM interaction with a particular emphasis on the role of TAMs in tumor immunity.

The review by Arrieta et al. [1] explores the origins of the various myeloid populations within GBM tumors. This includes the largely ignored macrophage populations in the CNS, for example, specialized subsets of macrophages which exist at the CNS borders including the perivascular, leptomeningeal, choroid plexus, and within the dural niches. The review also attempts to clarify which myeloid cells within the glioma tumor are a result of cancer-driven “emergency” myelopoiesis (i.e., myeloid-derived suppressor cells, MDSCs) and which myeloid cells are recruited locally from the brain which exist at a steady state within the CNS (i.e., microglia).

Macrophages within the glioma tumor have been shown to limit the effectiveness of oncolytic viral therapy. Blitz et al. [2] provide a comprehensive summary of different oncolytic viruses designed and tested in glioma pre-clinical models. It is noted that macrophages within tumors play opposing roles with respect to oncolytic viral therapy effectiveness. On the one hand, “M1”-type macrophages within tumors are desirable as they produce strong anti-viral cytokines and interleukins such as IL-12, and this generally benefits oncoviral therapy efficacy as well as the repolarization of the tumor microenvironment. On the other hand, macrophages are also responsible for clearing viruses to the tumor before the effective infection and killing of glioma cells can occur. Strategies to mitigate the anti-viral function of TAMs are discussed.

The review by Codrici et al. [3] is an overview of immune cell types within the glioma microenvironment with a specific focus on the chemokine system of small secreted proteins and their receptors. The role of chemokines in promoting immune cell recruitment to tumors and subsequent effects on glioma proliferation and angiogenesis is covered in detail. Additionally, a discussion of how the regulation of chemokines could potentially impact cell therapy, such as CAR-T and CAR-NK, for the treatment of glioma is included in this review.

Ma et al. [4] comprehensively describe the molecular players involved in paracrine interaction and signaling between glioma cells and TAMs. The difficulty of categorizing TAMs into distinct M1 or M2 phenotypes is noted due to the lack of consistent marker expression. The population of TAMs evolves with GBM progression. 

The review by Mohapatra et al. [5] covers a lot of ground with respect to the role of innate immunity in GBM. Interestingly, the authors describe the expression of “R-loops”, which are triple-stranded nucleic acid composed of RNA-DNA hybrids and non-template single-stranded DNA. The R-loops are postulated to exit the nucleus and engage cGAS and TLR3 in the cytoplasm to trigger an innate immune response.

Sharpe et al. [6] conducted an in silico analysis of GBM transcriptome databases and discovered a potential role for reproductive-associated regulatory T-cells (TREGs) in GBM tumors. This is significant as TREGs, along with pro-tumoral myeloid cells (such as MDSCs and M2 TAMs), are largely responsible for generating an immunologically “cold” tumor microenvironment. This study shows that GBM could express reproductive cell antigens such as GLUT14 to generate TREG infiltration and subsequent immunological tolerance. 

The final paper in this special edition is by Zeren et al. [7] and represents work from our laboratory wherein we show dependence on the chemokine receptor CCR1 for TAM-stimulated glioma invasion. We utilized pharmacological antagonists specific to CCR1 in glioma/macrophage coculture invasion assays in vitro. It is also noted that TAMs are induced to express high levels of CCR1 and its principal ligand, CCL3. It appears that the induction of CCR1 is at least partially dependent on CSF-1 signaling, as the pharmacological inhibition of CSF-1R attenuated CCR1 mRNA expression. Taken together, this suggests CCR1 might represent a more TAM-specific target as its expression is elevated within GBM tumors.

## References

[1] Arrieta V.A., Najem H., Petrosyan E., Lee-Chang C., Chen P., Sonabend A.M., Heimberger A.B. (2021). The Eclectic Nature of Glioma-Infiltrating Macrophages and Microglia. Int. J. Mol. Sci..

[2] Blitz S.E., Kappel A.D., Gessler F.A., Klinger N.V., Arnaout O., Lu Y., Peruzzi P.P., Smith T.R., Chiocca E.A., Friedman G.K. (2022). Tumor-Associated Macrophages/Microglia in Glioblastoma Oncolytic Virotherapy: A Double-Edged Sword. Int. J. Mol. Sci..

[3] Codrici E., Popescu I.D., Tanase C., Enciu A.M. (2022). Friends with Benefits: Chemokines, Glioblastoma-Associated Microglia/Macrophages and Tumor Microenvironment. Int. J. Mol. Sci..

[4] Ma J., Chen C.C., Li M. (2021). Macrophages/Microglia in the Glioblastoma Tumor Microenvironment. Int. J. Mol. Sci..

[5] Mohapatra S., Cafiero J., Kashfi K., Mehta P., Banerjee P. (2023). Why Don’t the Mutant Cells That Evade DNA Repair Cause Cancer More Frequently? Importance of the Innate Immune System in the Tumor Microenvironment. Int. J. Mol. Sci..

[6] Sharpe M.A., Baskin D.S., Jenson A.V., Baskin A.M. (2021). Hijacking Sexual Immuno-Privilege in GBM-an Immuno-Evasion Strategy. Int. J. Mol. Sci..

[7] Zeren N., Afzal Z., Morgan S., Marshall G., Uppiliappan M., Merritt J., Coniglio S.J. (2023). The Chemokine Receptor CCR1 Mediates Microglia Stimulated Glioma Invasion. Int. J. Mol. Sci..

